# Hypoxia Preconditioned Serum (HPS) Promotes Osteoblast Proliferation, Migration and Matrix Deposition

**DOI:** 10.3390/biomedicines10071631

**Published:** 2022-07-07

**Authors:** Jun Jiang, Lynn Röper, Sarah Alageel, Ulf Dornseifer, Arndt F. Schilling, Ektoras Hadjipanayi, Hans-Günther Machens, Philipp Moog

**Affiliations:** 1 Experimental Plastic Surgery, Clinic for Plastic, Reconstructive and Hand Surgery, Klinikum Rechts der Isar, Technische Universität München, D-81675 Munich, Germany; junqing.jiang@mri.tum.de (J.J.); lynn.roeper@mri.tum.de (L.R.); sarah.alageel@mri.tum.de (S.A.); e.hadjipanayi@googlemail.com (E.H.); 2 Department of Plastic, Reconstructive and Aesthetic Surgery, Isar Klinikum, D-80331 Munich, Germany; ulf.dornseifer@isarklinikum.de; 3 Department of Trauma Surgery, Orthopedics and Plastic Surgery, Universitätsmedizin Göttingen, D-37075 Göttingen, Germany; arndt.schilling@med.uni-goettingen.de

**Keywords:** peripheral blood cells, blood-derived therapy, hypoxia, osteogenesis, angiogenesis, hypoxia preconditioned serum, bone, osteoblasts, fracture healing, non-union, delayed union, regenerative medicine

## Abstract

Interest in discovering new methods of employing natural growth factor preparations to promote bone fracture healing is becoming increasingly popular in the field of regenerative medicine. In this study, we were able to demonstrate the osteogenic potential of hypoxia preconditioned serum (HPS) on human osteoblasts in vitro. Human osteoblasts were stimulated with two HPS concentrations (10% and 40%) and subsequently analyzed at time points of days 2 and 4. In comparison to controls, a time- and dose-dependent (up to 14.2× higher) proliferation of osteoblasts was observed after 4 days of HPS-40% stimulation with lower lactate dehydrogenase (LDH)-levels detected than controls, indicating the absence of cytotoxic/stress effects of HPS on human osteoblasts. With regards to cell migration, it was found to be significantly faster with HPS-10% application after 72 h in comparison to controls. Further osteogenic response to HPS treatment was evaluated by employing culture supernatant analysis, which exhibited significant upregulation of OPG (Osteoprotegerin) with higher dosage (HPS-10% vs. HPS-40%) and longer duration (2 d vs. 4 d) of HPS stimulation. There was no detection of anti-osteogenic sRANKL (soluble Receptor Activator of NF-κB Ligand) after 4 days of HPS stimulation. In addition, ALP (alkaline phosphatase)-enzyme activity, was found to be upregulated, dose-dependently, after 4 days of HPS-40% application. When assessing ossification through Alizarin-Red staining, HPS dose-dependently achieved greater (up to 2.8× higher) extracellular deposition of calcium-phosphate with HPS-40% in comparison to controls. These findings indicate that HPS holds the potential to accelerate bone regeneration by osteogenic promotion of human osteoblasts.

## 1. Introduction

Osteoporotic fractures are a major health issue and an economic burden [1]. The fracture incidence has steadily risen in the last 30 years worldwide, reaching 178 million cases in 2019 [2]. In Germany, fractures belong to the top 10 pathologies that lead to hospitalization (and are within the top three, when narrowing down to female patients over 65 years of age) [3]. The annual costs of fractures to the German healthcare system alone were estimated at 2.4 billion euros in 2009 [4]. When fractures require an operative treatment, postoperative complications such as non-union and delayed union are frequent in elderly patients and can be accompanied by joint stiffness, muscular atrophy or reflex sympathetic dystrophy [5]. Particularly hip fractures and their complications can have a substantial impact on the quality of life, as well as mid- and long-term function [6].

The repair of fractured bone tissue proceeds in the sequence of four stages, originally described by McKibbin: hematoma formation, inflammation, formation of callus and finally remodeling [7]. It follows an organized pattern of biological interactions between inflammatory, vascular and osteoprogenitor cell types to prepare the fracture site for its consolidation, restore vascularity and rebuild stable mechanics [8]. This process is critically dependent on the generation of gradients of biomolecules within the tissue. During fracture hematoma formation, the disruption of blood supply leads to a significant drop in oxygen availability, which induces changes in gene expression in osteoprogenitor cells, thus promoting their proliferation and formation of extracellular matrix through modulation of local and systemic cytokines, chemokines and growth factors [9,10]. Control of vascularization via Vascular Endothelial Growth Factor (VEGF) has been identified as a crucial key process in this context. Inhibition of VEGF severely impairs bone regeneration and leads to non-union formation [11,12]. In contrast, VEGF treatment successfully promotes fracture healing through pleiotropic effects, for example, proliferation and chemotaxis of osteoblasts, osteoclasts and chondrocytes [13,14]. Other cytokines and growth factors that have been shown to promote differentiation and proliferation of osteoblasts, as well as the calcification processes include OPG (Osteoprotegerin) [15], Osteocalcin [16], IGF-2 [17], bFGF [18] and BMP-2/-6/-7 [18,19,20]. In the clinical setting, single growth factor delivery by topical treatment, for example, BMP-2 and BMP-7, has been attempted in open tibial fractures and lumbar spine fusion, however at very high costs and with an unclear response rate [21]. A more complete and physiological growth factor preparation that is able to more accurately mimic the natural regeneration processes could be of larger clinical benefit.

Indeed, the current gold standard treatment of malunions by autologous bone grafting and mesenchymal stem cell therapy obtained from bone marrow aspirates partly relies on a multitude of biomolecules produced by the transplanted tissue [8]. Unlike these methods, which are burdened by donor site morbidity and high cost, we previously demonstrated the feasibility of using a simpler approach, specifically blood-derived secretomes, to promote tissue regeneration [22,23,24,25,26,27,28,29,30,31,32,33,34]. Peripheral blood cells (PBCs) are ideal providers of physiological regenerative signals which can be obtained on-demand using specific stress-induced treatment (e.g., hypoxia) [22,23,24,25,26,27,28,29,30,31,32,33,34]. In this context, we developed a novel method of extracorporeal hypoxia-adjusted in vitro preconditioning, which is based on incubating PBCs within a self-regulated low-oxygen microenvironment [23,24,25,29]. During this hypoxic stimulation, PBCs produce and release biomolecules into the serum compartment (hypoxia preconditioned serum: HPS), which can be separated from blood cells through filtration. We have previously shown that growth factors, such as VEGF [25,30,31,35], bFGF [25,30,31,35], IL-8 [25,30,31,35], MMP-9 [25,30,31,35] are strongly upregulated in HPS compared to unstimulated serum, while anti-angiogenic factors such as TSP-1 [25,27,30,31] are downregulated. This qualifies HPS as a promising candidate for boosting self-regeneration capacity. Indeed, we confirmed in several in vitro experiments that different processes involved in tissue regeneration, that is, angiogenesis/lymphangiogenesis, fibroblast proliferation and migration, can be promoted by HPS stimulation [22,23,24,25,26,27,28,29,30,31,32]—even under pathological conditions such as diabetes mellitus and cardiovascular disease treated with anticoagulant therapy [33]. Recently, we also successfully demonstrated the possibility of topically applying HPS via a hydrogel-carrier system to accelerate dermal wound healing in vivo [34].

Since soft tissue regeneration and bone healing share similar biological mechanisms, which are consistently initiated by hypoxia and can be promoted through accelerated vascularization, cell migration and cell proliferation [7,8,9,10], we sought to examine the effect of HPS treatment on osteoblast metabolism in order to evaluate its bone regeneration potential. Thus, we investigated the effects of HPS on human osteoblasts in vitro using two concentrations (10% and 40%) after 2 and 4 days of treatment. Parameters analyzed included cell proliferation and migration as well as proteomic analysis and ossification assays. Our findings demonstrate the upregulation of osteogenic processes in human osteoblasts treated by HPS, thus offering a platform for potentially engineering a topical therapy for bone fracture-healing.

## 2. Materials and Methods

### 2.1. Ethical Approval

This study was conducted per the Declaration of Helsinki and the approval of the ethics committee of the Technical University Munich, Germany (File Nr.: 307/17 S, date of approval: 29 August 2017). Informed consent was obtained from all blood donors involved in the study.

### 2.2. Production of Hypoxia Preconditioned Serum (HPS)

Production of HPS followed a protocol previously described by Hadjipanayi et al. [30]. The donor-selection criteria included: 10 healthy human donors (three females/seven males) with an age distribution ranging from 25 to 31 years, excluding smokers, pregnant donors, donors with systemic inflammatory diseases and donors with any oral medication in the last 6 weeks. For HPS preparation, peripheral venous blood was drawn, under sterile conditions, from each individual, and was collected into separate 30 mL polypropylene syringes (Omnifix^®^, B Braun AG, Melsungen, Germany). Then, 5 mL of air was drawn into each syringe through a 0.2 µm filter (Sterifix^®^, B Braun AG, Melsungen, Germany), with the plunger fully withdrawn. The sealed syringes were placed upright in an incubator at (37 °C/5% CO_2_) and incubated for 4 days. Instead of using a hypoxic incubator, we allowed cell-mediated O_2_ consumption to automatically generate local pericellular (i.e., surrounding the cell layer) hypoxia (~1% O_2_) within the blood-containing chamber. After incubation, the blood was separated as a result of sedimentation into three layers (serum, clot and red blood cell component). The top layer (HPS) was subsequently filtered (Sterifix^®^, B Braun AG, Melsungen, Germany) into a new syringe, thus eliminating cellular debris. In the next step, the cell-free HPS obtained from the 10 donors was pooled and stored at −80 °C until experimental testing (for a maximum of 3 months). Frozen and stored HPS secretomes (up to 3 months) have been previously assessed using VEGF-protein measurements and angiogenesis assays and exhibited no negative impact on the freeze-storage process in terms of angiogenesis although VEGF-protein levels were detected less [30,31].

### 2.3. Cell Culture

Human osteoblasts from three different donors (*n* = 3) were purchased from PromoCell (PromoCell GmbH, Heidelberg, Germany). Cryopreserved cells were thawed and cultured in Osteoblast Growth Medium (PromoCell GmbH, Heidelberg, Germany) containing Growth Medium Supplement Mix (PromoCell GmbH, Heidelberg, Germany) and 1% Antibiotic/Antimycotic Solution (ab/am) (Antibiotic/Antimycotic Solution, Amphotericin B 25.00 μg/mL, Penicillin 10,000 Units/mL, Streptomycin 10.00 mg/mL, Capricorn Scientific GmbH, Ebsdorfergrund, Germany) and placed into an incubator at 37 °C and 5% CO_2_. All ensuing experiments were carried out at the 7–8th cellular passages. For cell culture experiments, HPS was thawed and brought to a final concentration of 10% and 40% using DMEM (PAN BIOTECH, Aidenbach, Germany), 2% FCS (Biochrom GmbH, Berlin, Germany) and 1% ab/am. Control media contained only DMEM, 2% FCS and 1% ab/am (=culture media). The concentration of 2% FCS was determined in a preliminary experiment by using multiple FCS concentrations (2%, 5% and 10%) to ensure the viability of the cells by measuring cell counts and LDH (lactate dehydrogenase) for 4 days. Culture media in all experiments were provided fresh at the beginning of each test and were not changed over the time course of the experiment.

### 2.4. Cell Count Measurements

Cells were washed with PBS, trypsinized with Trysin/EDTA (Trypsin/EDTA Solution 0.25%/0.02% in PBS, Biochrom GmbH, Berlin, Germany), resuspended in 400 μL of DMEM/10%FCS and counted by an automated CASY cell counter (Roche, Mannheim, Germany). The CASY counter technology combines particle identification using resistance measurement with a pulse area analysis based on a digital pulse processing technology. Results of cell counts are given as cells per mL.

### 2.5. Lactate Dehydrogenase (LDH) Assay

Analysis of cell viability/cell toxicity was performed by quantitative measurement of lactate dehydrogenase (LDH)-activity. LDH is an enzyme located in the cytoplasm of cells. In the event of cell death or damage, it is released into the extracellular space. To investigate the amount of cell death in our experiments, osteoblasts were seeded at 30,000 cells/well into a 24-well plate and incubated for 2 and 4 days with culture media (DMEM, FCS 2%, ab/am 1%) containing HPS-10%/-40% and culture media-only as control group. Then, 100 μL of the cell culture supernatant was transferred into a 96-well plate. Commercially available Cytotoxicity Detection Kit LDH (Hoffmann-La Roche, Basel, Switzerland) was used per the manufacturer’s protocol. Pure HPS-10%, HPS-40% and control medium were used as blank controls. Optical densities (ODs) were then measured using Mithras LB 940 Multimode Microplate Reader (Berthold Technologies GmbH & Co. KG, Bad Wildbad, Germany) at a wavelength of 490 nm and a reference wavelength of 600 nm. Results of ODs are normalized by cell counts given in Section 2.4.

### 2.6. OPG and sRANKL Protein Quantification (ELISA)

Enzyme-linked immunosorbent assay (ELISA) was utilized to quantify the amount of Osteoprotegerin (OPG) and sRANKL (soluble Receptor Activator of NF-κB Ligand) protein produced by the cells. Accordingly, osteoblasts were seeded at 30,000 cells/well into a 24-well plate and incubated for 2 and 4 days with culture media (DMEM, FCS 2%, ab/am 1%) containing HPS-10% and HPS-40% final concentration and culture media-only as control group. At the indicated time points, the supernatant was harvested and stored at −20 °C. ELISAs were then performed according to the manufacturer’s protocols (OPG: Catalog # DY805; RANKL: Catalog # DY626; R&D Systems, Minneapolis, MN, USA). Optical density was measured using Mithras LB 940 Multimode Microplate Reader (Berthold Technologies GmbH & Co. KG, Bad Wildbad, Germany) at 450 nm wavelength. Cell supernatant was diluted at 1:60 in culture media to measure OPG with the above-mentioned ELISA kit.

### 2.7. Cell Migration Assay

Osteoblast migration assay was performed using culture-inserts u-dishes with a cell-free gap of 500 µm as described by the manufacturer (ibidi GmbH, Gräfelfing, Germany). In short, 70 µL of osteoblast suspension solution (3 × 10^5^ cells) reconstituted in culture media (DMEM, FCS 2%, ab/am 1%) was seeded into the inner well of the u-dish of a 24 well plate and incubated at 37 °C and 5% CO_2_ overnight. On the next day, the supernatant and migration chamber inserts were removed, unattached cells were rinsed off, and the osteoblasts were incubated with culture media (DMEM, FCS 2%, ab/am 1%) containing HPS-10% and HPS-40% final concentration and culture media-only as a control group. Osteoblast migration was determined by microscopic imaging at 0 h, 24 h, 48 h, 72 h and 96 h. Culture media were not changed over the time course of the experiment. The percentage of cell-free areas was quantified using the image analysis “Wimscratch tool” (Wimasis, Munich, Germany).

### 2.8. Alkaline Phosphatase (ALP) Activity Assay

Alkaline phosphatase (ALP) was quantified by a colorimetric enzymatic assay (Alkaline Phosphatase Assay Kit; Abcam, Cambridge, United Kingdom) by using the supernatant of the cell migration assay at day 4 (=96 h). Para-nitrophenyl-phosphate (pNPP) was used as the substrate (this turns yellow due to dephosphorylation by ALP). Briefly, 80 µL of supernatant samples, 50 µL of a 5 mM pNPP solution and 10 µL of the ALP enzyme were added subsequently to the 24-well plate. The plate was then incubated for 1 h at room temperature (=22 °C), deprived of light. Finally, 20 µL of stop solution was added, and the plates were measured at 405 nm using the Mithras LB 940 Multimode Microplate Reader (Berthold Technologies GmbH & Co. KG, Bad Wildbad, Germany). All results are expressed as absorbance recorded per minute (enzyme activity).

### 2.9. Alizarin Red Mineralization Assay

Alizarin red staining was used to determine osteoblast mineralization through calcium detection using the plates of the cell migration assay on day 4 (=96 h). For the staining protocol, 0.5 g Alizarin-Red Staining Solution (Sigma-Aldrich, St. Louis, MO, USA) was dissolved in 100 mL distilled water. The dye’s pH was brought to a value of 4.2 using ammonium hydroxide (Ammonium hydroxide solution 20–30%, Sigma-Aldrich, St. Louis, MO, USA) and hydrochloric acid (Hydrochloric acid Rotipuran^®^, Carl Roth GmbH + Co. KG, Karlsruhe, Germany). Then, the supernatant of the wells was removed and the cells were washed with 1 mL PBS. To fix the cells, 500 µL of 3.7% formaldehyde (formaldehyde—solution 3.5–3.7% buffered 1 L, Otto Fischer GmbH & Co. KG, Saarbrücken, Germany) was added per well and the cells were incubated at room temperature (=22 °C) for 5 min. After the formaldehyde was removed, 600 µL of Alizarin red solution was added to each well and incubated for 5 min at room temperature (=22 °C). Upon incubation termination, the wells were washed with distilled water. Photographs were taken with light microscopy and analyzed with ImageJ software (NIH, version 1.53, Bethesda, MD, USA). Using ImageJ CIELAB color filtering we were able to measure the area of the Alizarin red stain. The following settings were used (numbers indicate the minimum and maximum values while letters in brackets indicate the filter type: P—pass; S—stop): L* 0/255 (P), a* 0/130 (S), b* 0/255 (P). For each group (HPS-10%, HPS-40% and control), we used three osteoblast donors, all run in triplicates.

### 2.10. Statistical Analysis

Data sets were analyzed by one-way analysis of variance (ANOVA), with subsequent comparisons using Tukey’s post-hoc analysis, when one independent variable is present. In the case of repeated measures with two independent variables, a two-way repeated-measures ANOVA with Tukey’s multiple comparisons test was applied. All values are expressed as means ± standard error of the mean (SEM). A value of *p* < 0.05 was considered statistically significant (* *p* < 0.05, ** *p* < 0.01, *** *p* < 0.001, **** *p* < 0.0001).

## 3. Results

### 3.1. Hypoxia Preconditioned Serum (HPS) Promotes the Proliferation and Viability of Human Osteoblasts

**Figure 1:**
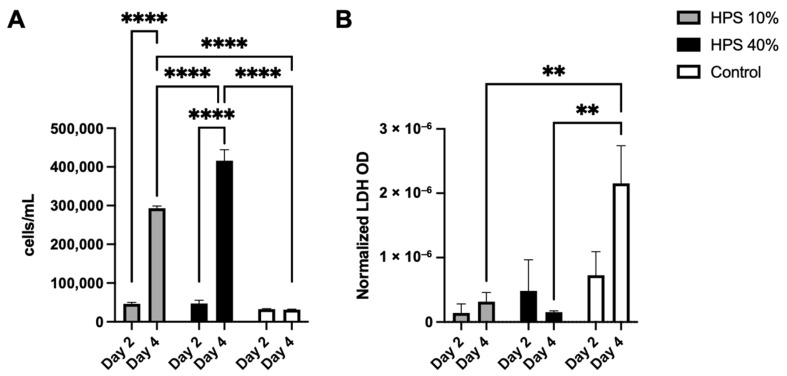
Cell proliferation and cytotoxic effect of hypoxia preconditioned serum (HPS) on human osteoblasts. (**A**) Cell counts of human osteoblasts with HPS-10% and HPS-40% stimulation compared to controls. Both HPS concentrations promote cell proliferation of human osteoblasts in a time- and dose-dependent manner. (**B**) Lactate dehydrogenase (LDH) assay: optical density (OD) normalized per cell. Cell cytotoxicity assessed by measuring LDH values was significantly lower after 4 days of HPS stimulation compared to controls (*p* < 0.01). Two-way repeated-measures ANOVA with Tukey’s multiple comparisons test. Data points are means ± SEM, *n* = 3. ** *p* < 0.01, **** *p* < 0.0001.

In the first series of experiments, we sought to examine the effects of HPS stimulation with regard to proliferation, viability and cytotoxicity of human osteoblasts. With the use of a fully automated cell counter, we determined the proliferative effects of two different HPS concentrations (10% and 40%) after 2 and 4 days of stimulation. Here, we found up to 1.4× increase in cell number on day 2 and up to 14.2× increase on day 4 with HPS-40% stimulation compared to controls (Figure 1A). Human osteoblast cell number observed with HPS-40% stimulation was also significantly higher than HPS-10% on day 4 (*p* < 0.0001), indicating a dose-dependent proliferative promotion by HPS. Moreover, both HPS-10% and HPS-40% stimulation resulted time-dependently in a higher osteoblast proliferation with longer HPS stimulation (2 days vs. 4 days; *p* < 0.0001). In the next step, we determined cell cytotoxic effects of HPS stimulation by LDH assay. Here, very low values of LDH (normalized per cell) were detected in all conditions with HPS stimulation (Figure 1B), specifically LDH release was comparable to the control group at day 2. Interestingly, cytotoxicity did not appear to increase at day 4 in the HPS groups, since LDH was significantly lower in both HPS-10% and HPS-40% compared to controls (*p* = 0.009 and *p* = 0.005 respectively).

### 3.2. Hypoxia Preconditioned Serum (HPS) Promotes Human Osteoblast Cell Migration

**Figure 2:**
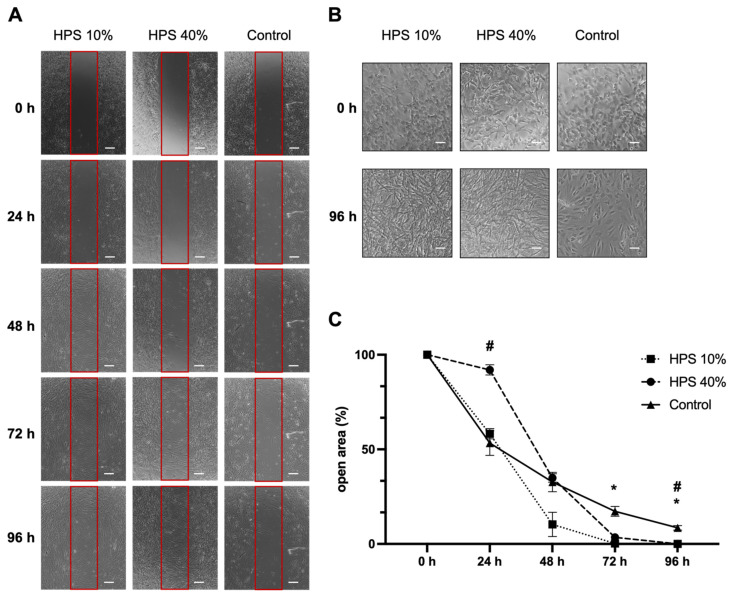
Osteoblast migration is accelerated by hypoxia preconditioned serum (HPS) stimulation. (**A**) Representative microscopic photographs of scratch assay with human osteoblasts (*n* = 3) stimulated by HPS-10% and HPS-40% compared to controls taken at 24 h intervals over a 96 h period. Red boxes indicate the initially standardized open area. Scale bar = 200 μm. (**B**) Image panel showing two-fold magnifications in HPS-10%, HPS-40% and controls at 0 h and 96 h demonstrating changes of cell shape from cuboid to elongated osteoblasts in the HPS treated groups. Scale bar = 100 μm. (**C**) Plot showing closure of the open area (residual area in % of the full area) calculated by image analysis of the digital photographs depicted in (**A**). Asterisk (*) indicates statistical significance between HPS 10% and controls, hash (#) indicates statistical significance between HPS-40% and controls. Two-way repeated-measures ANOVA with Tukey’s multiple comparisons test. Data points are means ± SEM, *n* = 3. * *p* < 0.05; # *p* < 0.05.

Osteoblast migration is one of the key steps of the bone formation process and fracture repair [36,37]. Therefore, we assessed the migration of osteoblasts using a scratch assay by monitoring a denuded open area (in vitro fracture model) with HPS-10% and HPS-40% application compared to controls (Figure 2A). Interestingly, we observed a slower migration of osteoblasts after 24 h with higher concentration (HPS-40%) than HPS-10% (open area: 92 ± 2.7% vs. 58 ± 2.7%, *p* = 0.002) and controls (open area: 92 ± 2.7% vs. 53 ± 6.5%, *p* = 0.03). Nonetheless, with longer culture duration, HPS-10% and HPS-40% accelerated closure of the open area more rapidly, particularly HPS-10%, which resulted in significantly smaller open area after 72 h compared to controls (0.2 ± 0.03% vs. 17.2 ± 2.60%, *p* = 0.04) and HPS-40% (0.2 ± 0.03% vs. 3.5 ± 0.35%, *p* = 0.02). HPS-40%-treated osteoblasts reached a significantly smaller open area after 96 h compared to controls (0.03 ± 0.03% vs. 8.50 ± 1.22%, *p* = 0.04). In summary, the open area closed the fastest with HPS-10% stimulation after 72 h, whereas controls did not appear to close even after 96 h. Furthermore, we discovered dose-dependent effects between HPS-10% and HPS-40%, which places HPS-10% favorably with regard to cell migration over a 96 h time frame (*p* = 0.002) (Figure 2C). Interestingly, in terms of cell morphology, we found that both HPS-10% and HPS-40%-treated osteoblasts started to turn from cuboid-shaped into elongated/spindle-shaped cells after 24 h, this change became more apparent after 96 h, whereas controls were mostly dominated by cuboid-shaped osteoblasts after 96 h (Figure 2B).

### 3.3. Hypoxia Preconditioned Serum (HPS) Promotes Osteogenic Effects by Osteoprotegerin (OPG) Secretion

Osteogenic and osteoclastic effects were determined by measuring culture supernatant cytokines Osteoprotegerin (OPG) and soluble Receptor Activator of NF-κB Ligand (sRANKL) after 2 and 4 days of HPS-10% and HPS-40% stimulation.

**Figure 3:**
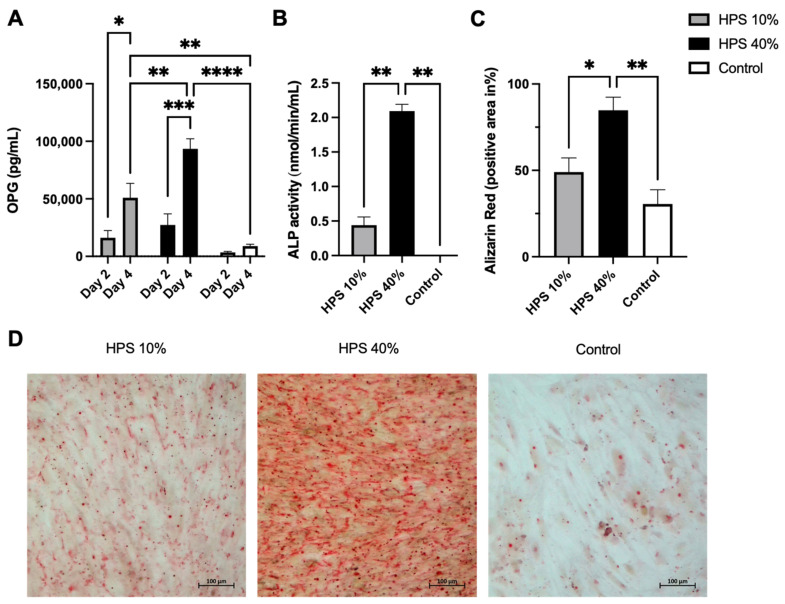
Hypoxia preconditioned serum (HPS) enhances osteogenic effects by upregulation of OPG secretion and ossification in human osteoblasts. (**A**) Quantitative measurement of Osteoprotegerin (OPG) levels in culture supernatants after 2 and 4 days of HPS-10%/-40% stimulation of human osteoblasts compared to controls. Time- and dose-dependent upregulation of OPG secretion was observed with HPS stimulation. Two-way repeated-measures ANOVA with Tukey’s multiple comparisons test. Data points are means ± SEM, *n* = 3. * *p* < 0.05, ** *p* < 0.01, *** *p* < 0.001, **** *p* < 0.0001. (**B**) Alkaline phosphatase (ALP) activity. Following 4 days of HPS stimulation, human osteoblasts were analyzed for ALP activity, which was higher with the higher dose of HPS-40% stimulation compared to HPS-10% and controls. (**C**) Quantitative Alizarin red measurement by digital photograph analysis. Calcification was elevated with higher HPS concentration (HPS-40%) compared to HPS-10% and controls. (**B**,**C**) One-way ANOVA with Tukey’s multiple comparisons test. Data points are means ± SEM, *n* = 3. * *p* < 0.05, ** *p* < 0.01. (**D**) Representative high-power fields of Alizarin red staining of HPS-10%, HPS-40% stimulated osteoblasts and controls. Scale bar = 100 μm.

OPG is a soluble tumor necrosis factor receptor (TNFRSF11B) secreted by osteoblasts. It functions as a decoy receptor for RANKL thus inhibiting the binding and activation of RANK on osteoclast precursors [38], which ultimately prevents bone resorption [39]. OPG is therefore perceived as a key anti-osteoclastogenic cytokine and the ratio of OPG/RANKL modulates bone remodeling and bone mass [39]. It has to be noted that OPG is a marker of osteoblast differentiation and is increasingly expressed in matured osteoblasts [40]. Here, OPG was detected in greater concentration with a longer and higher dose of HPS stimulation (Figure 3A), indicating that HPS may also promote osteoblast differentiation. We were able to detect up to a 7.9-fold increase in OPG secretion on day 2 and up to a 10.5-fold increase on day 4 with HPS-40% stimulation compared to controls. OPG secretion by HPS-40% stimulation was also significantly higher than by HPS-10% stimulation on day 4 (*p* = 0.006), indicating a dose-dependent upregulation by HPS. In addition, both HPS-10% and HPS-40% stimulation resulted in a time-dependent manner in higher OPG supernatant levels with longer HPS stimulation duration (4 days vs. 2 days) (HPS-10%: *p* = 0.013, HPS-40%: *p* = 0.0004).

Soluble Receptor Activator of NF-κB Ligand (sRANKL) protein measurements were performed from the same above-mentioned supernatants. RANKL is perceived as osteoclastogenic in bone metabolism and is a membrane-bound protein [39]. It can be cleaved by matrix metalloproteases and released into the extracellular space as soluble RANKL (sRANKL) [39]. Although evidence was presented that the formation of osteoclasts requires contact with osteoblast lineage cells with membrane-bound RANKL (mRANKL), sRANKL was able to promote osteoclast formation from precursors as well [41]. However, sRANKL protein was neither detected in the controls nor in the HPS-stimulated supernatant by ELISA (data not shown). Hence, HPS does not appear to stimulate sRANKL protein production of human osteoblasts in the first 4 days of stimulation which would indicate no direct upregulation of osteoclastogenic effects within this time frame.

### 3.4. Hypoxia Preconditioned Serum (HPS) Promotes Alkaline Phosphatase (ALP) Activity and Ossification

In the final step, we investigated alkaline phosphatase (ALP) enzyme activity to determine the ossification capacity of HPS-stimulated human osteoblasts. It has to be noted that ALP is primarily expressed in matured osteoblasts [42]. Here, ALP was increasingly enhanced after 4 days of HPS stimulation (Figure 3B) which may indicate a higher differentiation of osteoblasts. HPS-40% stimulated osteoblasts displayed notably higher ALP activity than HPS-10% (2.09 vs. 0.44, *p* = 0.004) and controls (2.09 vs. 0.002, *p* = 0.004). To verify this result, we investigated levels of mineralized calcium nodule formation by Alizarin red staining. We used the same experimental setting from above and stained 4 days of HPS-stimulated osteoblasts and controls. Digital photographs were taken (Figure 3D) and analyzed by image analysis. Here, we demonstrate that ALP-activity correlates to the degree of calcium deposits in the same dose-dependent manner (Figure 3C). HPS-40% stimulated osteoblasts produced 2.8× more calcium nodules in comparison to HPS-10% (*p* = 0.04) and controls (*p* = 0.007). Therefore, HPS may promote ossification in a dose-dependent manner through ALP upregulation in human osteoblasts.

## 4. Discussion

Hypoxia preconditioned serum (HPS) is a new-generation blood-derived secretome that provides a therapeutic tool for bioactively supporting tissue repair by promoting angiogenesis/lymphangiogenesis, proliferation/migration of fibroblasts and dermal skin regeneration [22,23,24,25,26,27,28,29,30,31,32,34]. As the osteoblast can be considered a specialized fibroblast [43], we were intrigued about whether HPS has a similar impact on bone cells. Therefore, in this study, we employed HPS on human osteoblasts to examine its biological effects. Our investigations were able to demonstrate a substantial osteogenic response to HPS treatment via the promotion of proliferation, migration and differentiation of human osteoblasts. An upregulation of Osteoprotegerin (OPG) cytokine protein and alkaline phosphatase (ALP) enzyme activity was observed which suggests a possible activation of bone tissue production and higher matrix calcification both in vitro and in vivo [39,44]. Therefore, HPS holds the potential of promoting bone regeneration.

Osteoblast proliferation is recognized as a hallmark of fracture healing: Bone-lining cells are well-documented for the expansion of osteoprogenitor cell populations which appears to account for the formation of bone callus during bone repair [37]. Previous work in rodents has shown that cell proliferation in periosteal callus is elevated on day 2 and remains elevated until 14 days after a fracture incident [45,46]. Therefore, the first two weeks post-fracture are considered a critical period for cell proliferation during the healing process, while impaired cell proliferation during this period will lead to blunted callus formation, which may in turn result in atrophic non-union and pseudarthrosis [45,46]. In this study, HPS was shown to stimulate osteoblast proliferation up to 14.2× more than controls (Figure 1A). This effect was demonstrated dose- and time-dependently. Thus, greater cell proliferation was observed by a higher HPS concentration (HPS-10% vs. HPS-40%) and a longer stimulation period (2 d vs. 4 d). Cell cytotoxicity was measured with LDH assay, which confirmed low LDH levels that were comparable to controls on day 2 and were even considerably lower than controls on day 4 (Figure 1B), which suggests that HPS does not likely inflict cytotoxic effects on human osteoblasts and might even promote osteoblast viability. Consequently, HPS may support bone regeneration processes within the early stages of fracture healing.

A deliberate choice was made for testing the HPS concentrations of 10% and 40% in this dose-dependent investigation of osteogenic and cytotoxic effects since we had previously established the safety and effectiveness of these concentrations in several in vitro angiogenesis/lymphangiogenesis assays, fibroblast migration experiments and more recently, in an in vivo murine dermal skin regeneration model [25,26,28,29,30,31,32,33,34]. HPS dilutions are necessary to investigate the net effect of all growth factors in HPS since this depends on the balance of pro- and anti-angiogenic protein factors, the influence of the latter being diminished by dilution: Indeed, we had previously demonstrated greater angiogenesis with higher HPS dilutions [31]. Additionally, we preliminary tested HPS-1%, -5%, -10% and -40% in osteoblast proliferation, LDH-cytotoxicity and ALP-activity on day 2 and 4, which showed no considerable differences in HPS-1% and -5% compared to the control group (Figure S1). With regards to the choice of culture duration, at 4 days of HPS stimulation, osteoblasts reached over 95% confluency in the culture wells, thus a longer culture time point was not investigated since any residual effects would be too small to detect.

During bone remodeling and fracture repair, bone formation requires mature osteoblasts to deposit bone with remarkable spatial precision during their migration within the three-dimensional bone matrix [36]. Here, we demonstrate the elevated cell migration capacity of human osteoblasts treated with HPS using the scratch assay (Figure 2). Interestingly, the lower HPS concentration (HPS-10%) appears to outperform the higher concentration (HPS-40%) in the early stages of stimulation (24–72 h) and also overtake the cell migration speed of the control group after 24 h (Figure 2C). The gap closure in the scratch assay was notably earlier (after 72 h) with HPS-10%, compared to HPS-40% stimulation, which required 96 h. The poorer migration of the HPS-40%-stimulated osteoblasts in the first 24 h (Figure 2C) may have resulted from an excess supply of growth factors and cytokines, which seems to reach optimal migration capacity either through a four-fold dilution of HPS (as in HPS-10%), or partial degradation of hypoxia-induced cytokine proteins after 24 h (which then reaches similar gap closure speed to HPS-10%) (Figure 2C). Further investigations to assess the effects of higher dilutions of HPS on the migration capacity of the cells are to be conducted. In contrast to the benefit of higher HPS concentration on cell proliferation and viability mentioned earlier, it has to be evaluated which HPS concentration promotes bone healing more efficiently in an in vivo fracture model, provided that cell migration to the fracture site is a pre-requisite for initiation of the regenerative process and hierarchically may even more important than cell proliferation.

With regards to bone mass homeostasis through key regulation of the OPG/RANKL ratio, we found significant upregulation up to 10.5× of osteogenic OPG compared to controls (Figure 3A), but no detection of soluble RANKL (sRANKL) as an osteoclastogenic cytokine at least within 4 days of HPS stimulation. Apart from sRANKL, also membrane-bound RANKL (mRANKL) can stimulate osteoclast formation and maturation [47]. Indeed, the magnitude of the reduction in osteoclast number in mice lacking sRANKL was smaller than in mice lacking mRANKL indicating that mRANKL is more dominant and effective than sRANKL [48]. Thus, protein identification of mRANKL by immunohistochemical cell staining or expression evaluation via RANKL mRNA measurements is still to be performed in order to study the interaction of osteoblasts/osteoclasts and the OPG/RANK/RANKL-axis-modulation in the setting of HPS stimulation. Additionally, since the physiological RANKL protein level in human blood serum of approximately 250 pg/mL [49] was not detectable in the culture supernatants, RANKL is suggested to be degraded with time and was at least not reproduced as a soluble cytokine from osteoblasts during HPS stimulation. However, a more extensive investigation of the effects of HPS on bone mass homeostasis must be conducted using a model of larger complexity and including all cell types involved in bone regeneration, especially osteoclasts. Such a model would be possible in an in vitro co-culture system with osteoblasts/osteoclasts [50] or an ex vivo embryonic chick femur model [51].

Alkaline phosphatase (ALP) is an early marker of osteoblast differentiation and its increased expression is associated with the progressive maturing of osteoblasts [42]. It has a critical function in the formation of hard tissue and facilitates mineralization [44]. In turn, the mineralization process is important for the accumulation of Ca^2+^ and inorganic phosphate, which serve as components for the formation of hydroxyapatite, which buds from the outer membrane of osteoblasts [44]. Calcification can be detected through Alizarin red staining and has been used here to evaluate calcium-rich deposits by osteoblasts stimulated with HPS. In this study, we confirmed a significant dose-dependent upregulation of ALP which is also reflected in the higher dose-dependent extracellular calcium deposition by HPS-stimulated osteoblasts (Figure 3C,D). In the control group, osteoblasts showed almost no detectable ALP activity and areas without any Alizarin red staining (Figure 3C,D). However, both elevated ALP-activity and higher calcium detection through HPS stimulation must also be partly derived from higher cell counts, induced by HPS stimulation itself. In summary, considering the net effect of calcium production, we can postulate that HPS stimulates mineralization through both differentiation and cell proliferation of osteoblasts.

With respect to cell differentiation at a morphological level, it is not fully clear from these results which osteoblast cell type predominates post-HPS stimulation. Osteoblasts are cuboid cells that are reported as post-mitotic cells [52]. Pre-osteoblasts and osteoprogenitor cells have mitotic capacities but are described as elongated cells [52]. After analyzing the light microscopy images from the scratch assay timeline (Figure 2A), we have observed, at the 0 h-start point, homogeneously distributed cuboid-shaped osteoblasts (see magnifications in Figure 2B). Yet with further HPS stimulation, these osteoblasts appear to become more elongated and were prominent in the scratch gap in addition to the plated area. After 96 h (4 days), HPS-stimulated osteoblasts had a notably higher density than controls and appeared to develop more cell-cell contacts. At this stage, elongated cells were predominantly present at the scratch gap and plated area of HPS-stimulated osteoblasts, while osteoblasts of the control group showed mostly cuboidal-shaped cells. Given these observations, the question arises whether HPS stimulates the de-differentiation of osteoblast back to pre-osteoblasts, which may indeed explain their higher proliferation capacity under HPS stimulation. This remarkable plasticity in reverse differentiation is already known in the mesenchymal cell lineage and was demonstrated previously through alterations in the cell environment of osteoblasts [53]. Moreover, this de-differentiation capacity is also supported by the increased migration capacity of the HPS-stimulated cells, which is exclusively reported in pre-osteoblasts and osteoprogenitor cells rather than matured osteoblasts [36]. Nevertheless, the absolute number of matured osteoblasts must have been higher under HPS stimulation since OPG and ALP were considerably upregulated compared to controls (Figure 3A,B). This leads to the deduction of a possible co-existence of both matured osteoblasts and pre-osteoblasts/osteoprogenitor cells and indicates both differentiation and de-differentiation capacities of HPS. In addition to the above-mentioned upregulation of OPG and ALP through HPS stimulation, further investigation of osteoblast differentiation markers, for example, COL1a2 (collagen type 1 alpha 2), RUNX2 (Runt-related transcription factor 2) and Osteocalcin expression/staining, would be of benefit in the confirmation of the osteoblast differentiation and de-differentiation capacities of HPS. Indeed, we consider it as a limitation, that mRNA measurements could not be included in this study.

HPS presents a novel approach to the current gold-standard blood-derived therapy, namely platelet-rich plasma (PRP) [54]. The growth factor release in PRP relies on the activation of platelets which are concentrated by centrifugation to a supraphysiological level [55]. PRP recapitulates the hemostatic phase of the regeneration process to support the restoration of tissue integrity, unlike HPS, which harnesses all wound healing phases from hemostasis, inflammation to proliferation [56]. Bone regeneration with PRP application has become common in oral and maxillofacial surgery which targets the promotion of osseointegration after placement of dental implants and the enhancement of bone formation in autologous bone grafting [57,58,59,60]. Key growth factors involved in the accelerated bone regeneration observed with PRP include PDGF, TGF-beta, IGF, bFGF and VEGF [57,61]. These cytokines are proven to play a key role in chemoattraction/proliferation of osteoblasts and matrix ossification processes [13,14,17,18,36]. Similar to PRP, the method of hypoxic incubation of PBCs delivers a secretome which also includes the above-mentioned upregulation of key regenerative growth factors. However, HPS can be differentiated from PRP by the fact that it comprises “newly-produced” factors released by PBCs in response to hypoxia, rather than simply being a composition of passively released factors stored within platelets. Nevertheless, both blood-derived secretomes (PRP and HPS) are defined by a complex balance of pro- and anti-angiogenic proteins where the net effect is inherently difficult to estimate. For instance, we have previously observed a stronger angiogenic response to HPS in comparison to PRP, by examining microvessel formation and sprouting in endothelial cell and aortic ring cultures [31]. A comparable study investigated the effect of PRP and PRF (Platelet-rich fibrin: PRP, which contains leukocytes) on human osteoblast proliferation and cell migration which showed that PRP had a significantly lower proliferation effect than PRF and also failed to close the gap of the scratch assay after 72 h (40% vs. 100% closure) [61]. Arguably, this was caused by the addition of leukocytes in PRF, which seem to increase the growth factor release of TGF-beta, VEGF and PDGF [62]. These results were also confirmed by another group, which evaluated the beneficial effect of the leukocytes in PRF in comparison to PRP [63]. Based on these results, we believe that a superior osteogenic response may be obtained by using HPS, with comparable outcomes to PRF due to its resemblance in cell composition. A head-to-head comparison of the effects of HPS and PRP/PRF in a standardized experimental setup remains to be investigated.

In the clinical setting, HPS could be a promising candidate for localized application in fracture-healing therapy. Our group has previously engineered an integral HPS-fibrin-hydrogel system that releases protein factors at the site of application, thus allowing the gradual generation of spatiotemporal gradients of hypoxia-induced protein factors [24,25,30]. Fibrin-hydrogel carriers are easy to apply, either through injection or topical application and also have the advantage of potentially serving as scaffolds for cellular ingrowth and biological defect coverage [24,25,27,30]. Moreover, HPS–fibrin-hydrogels present an opportunity for a paradigm shift in the utilization of injectable compositions, away from that of inert biomaterials that primarily induce a foreign body reaction, towards biomimetic and bioactive scaffolds. In vivo HPS-hydrogel-mediated healing of dermal wounds has already been demonstrated by our group [34]. Further investigation is needed to validate the same healing efficacy in the field of bone regeneration.

It has to be noted, that some of the demonstrated effects of HPS on human osteoblasts may be, at least partly, derived from the serum component itself, besides the additional changes induced in its composition via hypoxic preconditioning (HPS). Human serum is considered to be an alternative substitute to the fetal calf serum (FCS), which we used in 2% concentration in the culture media in all of the groups. As mentioned earlier, we conducted a preliminary experiment to assess the effect of FCS concentration on osteoblasts proliferation and viability. There was no significant difference in these parameters tested for FCS concentration up to 10% (2%, 5%, 10%) until 4 days of osteoblast cultivation (data not shown). However, despite not having included a basal human serum-only control group, which is an admittable limitation of this study, the hereby demonstrated osteoblast differentiation with upregulation of OPG, ALP, Ca^2+^ deposition and cell migration cannot be simply explained by a higher serum concentration in HPS-10% and HPS-40% alone: on the contrary, a lower FCS concentration of 2% has been shown previously in vitro to generate a greater ALP upregulation and more Ca^2+^ deposits than 10% FCS [64]. In contrast, we found in additional experiments a dose-dependent increase of ALP with no significant effect up to HPS-5% and rising HPS concentrations thereafter (Figure S1C). Furthermore, the higher migration capacity observed with HPS-10% rather than HPS-40% stands against the higher proliferation capacity seen with HPS-40%. These findings cannot be easily explained by the effect of serum alone and therefore, the most probable origin of these differences lies in the effect of hypoxia-induced growth factors. Further extensive studies should be directed towards an experimental setting in which osteoblasts are investigated with the inclusion of a basal human serum-only group to exclusively demonstrate the effect of the hypoxia-induced secretome.

## 5. Conclusions

Our findings demonstrate the regenerative potential of hypoxia preconditioned serum (HPS) on human osteoblasts in vitro. Its osteogenic effect suggests that utilization of HPS therapy would be similarly effective in bone regeneration. However, advances in in vivo bone tissue engineering require further studies that will explore the complex interaction of a more encompassing set of cell types, for example, osteoclasts and blood-circulation-derived leukocytes, in order to more accurately analyze its overall effect on bone mass homeostasis.

## 6. Patents

Device-based methods for localized delivery of cell-free carriers with stress-induced cellular factors. (AU2013214187 (B2); 9 February 2017): Schilling Arndt, Hadjipanayi Ektoras, Machens Hans-Günther.

## Supplementary Materials

The following supporting information can be downloaded at: https://www.mdpi.com/article/10.3390/biomedicines10071631/s1, Figure S1: Analysis of osteoblast proliferation, LDH-cytotoxicity and ALP-activity in dependency of HPS concentration.

## Abbreviations

ALP	Alkaline phosphatase
BMP	Bone morphogenic protein
bFGF	Basic fibroblast growth factor
HPS	Hypoxia preconditioned serum
IGF	Insulin-like growth factor
IL-8	Interleukin-8
LDH	Lactate dehydrogenase
MMP-9	Matrix metalloproteinase-9
mRANKL	Membrane-bound Receptor Activator of NF-κB Ligand
OPG	Osteoprotegerin
PBC	Peripheral blood cells
PDGF	Platelet-derived growth factor
PRP	Platelet rich plasma
PRF	Platelet rich fibrin
RANK	Receptor Activator of NF-κB
RANKL	Receptor Activator of NF-κB Ligand
sRANKL	Soluble Receptor Activator of NF-κB Ligand
TGF-beta	Tumor growth factor-beta
TSP-1	Thrombospondin-1
VEGF	Vascular endothelial growth factor
