# Supplementary material for: Hypoxia Preconditioned Serum (HPS) Promotes Osteoblast Proliferation, Migration and Matrix Deposition
Source: Biomedicines. 2022 Jul 7;10(7):1631. doi: 10.3390/biomedicines10071631 (PMC9313157; doi:10.3390/biomedicines10071631)
Supplement: Supplementary file 1 [file biomedicines-10-01631-s001.zip › biomedicines-1782663-supplementary.pdf]

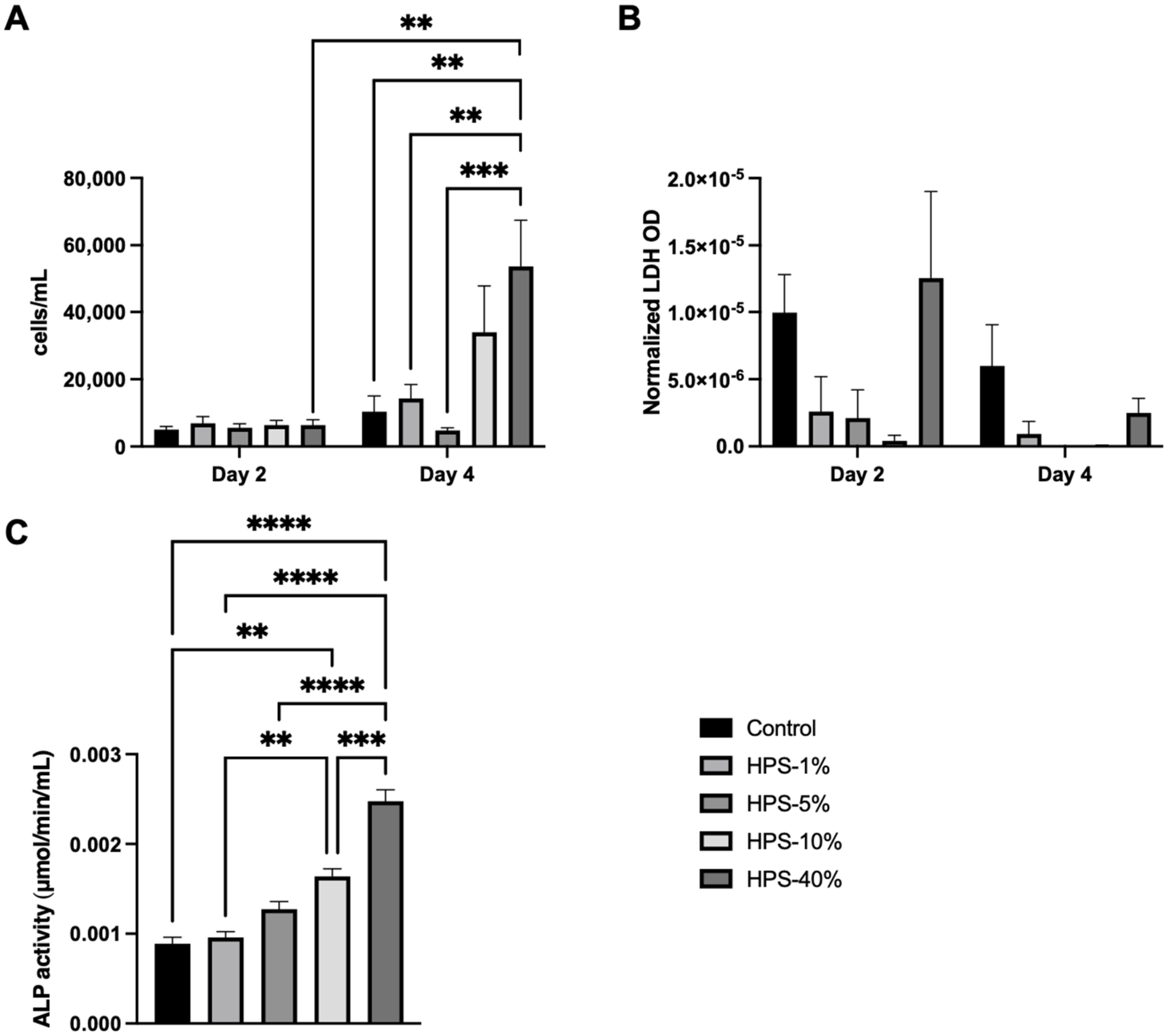

Figure S1. Analysis of osteoblast proliferation, LDH-cytotoxicity and ALP-activity in dependency of HPS-concentration. Osteoblasts seeded 5,000 per 24 well in HPS (diluted in culture media) in comparison to controls (culture media-only). **(A)** Cell counts of human osteoblasts on day 2 and day 4. **(B)** Lactate dehydrogenase (LDH) assay: optical density (OD) normalized per cell on day 2 and day 4. **(C)** Alkaline phosphatase (ALP) activity after 4 days of cultivation. (A)+(B): Two-way repeated-measures ANOVA with Tukey's multiple comparisons test. (C): One-way ANOVA with Tukey's multiple comparisons test. Data points are means ± SEM, n=3. **$p < 0.01$, ***$p < 0.001$, ****$p < 0.0001$.
